# A journey through chaos and calmness: experiences of mindfulness training in patients with depressive symptoms after a recent coronary event - a qualitative diary content analysis

**DOI:** 10.1186/s40359-018-0252-1

**Published:** 2018-09-13

**Authors:** Oskar Lundgren, Peter Garvin, Margareta Kristenson, Lena Jonasson, Ingela Thylén

**Affiliations:** 1 Crown Princess Victoria Children’s Hospital, Linköping, Sweden; 2 Division of Community Medicine, Department of Medical and Health Sciences, Linköping University, Linköping, Sweden; 3 Research and Development Unit in Region Östergötland, Linköping, Sweden; 4 Division of Cardiovascular Medicine, Department of Medical and Health Sciences, Linköping University, Linköping, Sweden; 5 Division of Nursing, Department of Cardiology and Department of Medical and Health Sciences, Linköping University, Linköping, Sweden

**Keywords:** Mindfulness based stress reduction, Depressive symptoms, Myocardial infarction, Unstable angina pectoris, Qualitative content analysis

## Abstract

**Background:**

Psychological distress with symptoms of depression and anxiety is common and unrecognized in patients with coronary artery disease (CAD). Efforts have been made to treat psychological distress in CAD with both conventional methods, such as antidepressant drugs and psychotherapy, and non-conventional methods, such as stress management courses. However, studies focusing on the experiences of mindfulness training in this population are still scarce. Therefore, the aim of this study was to explore immediate experiences of mindfulness practice among CAD patients with depressive symptoms.

**Methods:**

A qualitative content analysis of diary entries, written immediately after practice sessions and continuously during an 8-week long Mindfulness Based Stress Reduction course (MBSR), was applied.

**Results:**

Twelve respondents participated in the study. The main category: *a journey through chaos and calmness* captured the participants’ concurrent experiences of challenges and rewards over time. This journey appears to reflect a progressive development culminating in the harvesting of the fruits of practice at the end of the mindfulness training. Descriptions of various challenging facets of mindfulness practice – both physical and psychological - commonly occurred during the whole course, although distressing experiences were more predominant during the first half. Furthermore, the diary entries showed a wide variety of ways of dealing with these struggles, including both constructive and less constructive strategies of facing difficult experiences. As the weeks passed, participants more frequently described an enhanced ability to concentrate, relax and deal with distractions. They also developed their capacity to observe the content of their mind and described how the practice began to yield rewards in the form of well-being and a sense of mastery.

**Conclusions:**

Introducing MBSR in the aftermath of a cardiac event, when depressive symptoms are present, is a complex and delicate challenge in clinical practice. More nuanced information about what to expect as well as the addition of motivational support and skillful guidance during the course should be given in accordance with the participants’ experiences and needs.

**Trial registration:**

The trial was retrospectively registered in clinicaltrials.gov (registration number: NCT03340948).

**Electronic supplementary material:**

The online version of this article (10.1186/s40359-018-0252-1) contains supplementary material, which is available to authorized users.

## Background

Psychological distress, including symptoms of depression and anxiety, is common though often unrecognized in patients with coronary artery disease (CAD) [1, 2]. This is troublesome since recent studies have shown that psychological stress and distress could both worsen the disease process [3] and make it harder for the patients to deal with the complexities of life [4]. However, these psychosocial risk factors are modifiable and thereby feasible targets for preventive efforts and interventions [5]. Indeed, policy documents recommend tailored psychosocial interventions in cardiac rehabilitation [6], but in clinical reality awareness and initiative in this domain are still lacking [7]. Efforts have been made to treat psychological distress in CAD with both conventional methods; e.g. antidepressant drugs and psychotherapy [8–10] and non-conventional methods; e.g. stress management courses [11]. Although the first trials showed only modest effects [8] later trials have shown promising effects on symptoms of distress and a small protective secondary preventive effect on cardiac events from psychological interventions, as described in a Cochrane systematic review [12]. Furthermore, in a recently published prospective study, we showed that psychological resources, such as sense of mastery and high self-esteem, had protective cardiovascular effects [13]. An old method, that recently has found a renaissance in medicine aiming to strengthen psychological functioning, is mindfulness meditation.

### Mindfulness based interventions

Mindfulness based interventions (MBI:s) are a family of programmes that have been utilized in the treatment of psychological distress in different somatic diseases since the 1980’s [14]. Mindfulness training is most commonly delivered through one of the two related interventions Mindfulness Based Stress Reduction (MBSR), developed in a medical context [15] and Mindfulness Based Cognitive Therapy (MBCT), developed in a psychiatric context [16]. These 8-week long courses in mindfulness meditation and yoga have shown to generate robust improvements in perceived stress, quality of life, depressiveness and anxiety [14]. Our choice of investigating MBSR in the cardiac rehabilitation context was based on the evidence base for the suitability of this intervention for chronically somatically ill patients [14]. Kabat Zinn describes mindfulness as *paying attention, on purpose, in the present moment and as non-judgmentally as possible* [17]. Shapiro et al. [18] have refined this definition and clarified that it contains three interrelated parts; *intention, attention* and *attitude.* The third part has also been described as a very specific way to *relate* to experiences (with *equanimity*) that facilitates psychological well-being. This skill might also take longer time to cultivate than the intentional- and attentional facets [19].

The application of MBI:s in the field of cardiology is a recent endeavour [20]. Louks et al. [21] have recently shown that dispositional mindfulness is related to cardiac health and early trials have shown promising results in various cohorts of CAD patients [22, 23]. Although MBSR and MBCT are considered effective and safe treatments [14] and their plausible psychobiological mechanisms are discussed [24], large gaps still exist in our understanding of the potential and limitations of these methods. Mindfulness research is in its adolescence and it has been criticized for over-enthusiasm, vague definitions of key concepts, uncritical implementation in clinical practice, simplification of the complex psychobiological processes at work and a lack of convergence between classic and modern practices and concepts [25]. Furthermore, there are still unanswered questions regarding which patients benefit from these interventions, what represents an adequate dose of meditation training, how the practices translate into wholesome behaviours and how to reach and motivate those who are in most need of treatment. To address some of these remaining questions, it might be necessary to complement psychometric approaches e.g. questionnaires, with qualitative methods that can elucidate the rich inner experience of patients in ways psychometric self-report methods are not able to do. As far as our knowledge extends, only one study has investigated the experience of mindfulness training among CAD patients with psychological distress [26]. Griffiths et al. [26] interviewed 10 patients 6–12 weeks after MBCT and found five different themes that described participant’s responses; *development of awareness*, *group experience*, *commitment*, *relating to material* and *acceptance as an outcome*. There are, however, implicit methodological shortcomings in interviewing participants long after completion of the intervention, since recall difficulties might result in biased data [27]. Moreover, when collecting qualitative data over time, diaries have been suggested as a suitable data collection method to facilitate participant’s recall [28]. Therefore, in order to capture the immediate experience of mindfulness practice, it would be more fruitful to collect data in close proximity to the practice sessions. In order to study the potential benefits from and barriers to the practice of mindfulness meditation among CAD patients with elevated depressive symptoms, our aim was to explore participants’ immediate experiences of a MBSR course.

## Methods

### Study design

This qualitative study was conducted as an independent part of a larger study aimed at describing the feasibility and acceptability of the original 8-week MBSR program in patients with depressive symptoms after a recent CAD event (Lundgren O, Garvin P, Nilsson L, Tornerefelt V, Andersson G, Kristenson M, Jonasson L: Mindfulness based stress reduction for coronary artery diseasepatients: potential improvements in mastery and depressive symptoms, submitted). We applied a qualitative content analysis of participants’ diary entries, written immediately after practice sessions and continuously during the whole course. During a 10 month-period in 2012–2013, 193 patients, with a recent diagnosis of first time CAD event (i.e. myocardial infarction or unstable angina pectoris) were consecutively assessed for depressive symptoms 1 month after the event, when they came to a follow-up visit to their cardiac nurse. At this point in time, patients with transient psychological distress related to the event, who are known to have a better prognosis would have had a chance to recover [29]. Patients with elevated levels of depressive symptoms, defined as a score of 8 or higher on the Centre for Epidemiological Studies Depression Scale (CES-D) [30], were invited by letter to participate in an 8-week MBSR intervention. The intention was to recruit patients with psychological distress, including mild to moderate clinical depression. The 20-item CES-D scale was deemed suitable since it can assess a broad continuum of levels of depressive symptoms, from well-being over mild to severe levels of depression [31]. One criterion for exclusion was severe clinical depression (based on physician’s clinical judgment), since the latter might imply difficulties to complete MBSR. Furthermore, the inclusion of severely depressed patients, would have raised ethical- and methodological questions related to the use additional psychiatric treatment during the intervention, and the rationale would be weaker since this group is the only one where psychopharmacological treatment are known to be effective [32]. Other exclusion criteria were severe comorbidities, such as cancer, severe cognitive impairment, psychosis, serious personality disorder, alcohol or drug abuse and bipolar disease. If patients gave a positive response to the letter they were informed via phone about the 8-week MBSR course. Twenty-four participants started MBSR whereof 16 completed the course.

### The MBSR intervention

The MBSR intervention consisted of 8 weekly 2.5 h group sessions, and one silent all-day mini-retreat (6 h) in week 6 [17]. Group sessions were located to the University Hospital, and led by the first author (OL) of the study. The participants received CDs with guided instructions, as well as a workbook with reflection exercises and a diary (see below). Recommended practice time at home was 40 min, 6 days a week. The body scan exercise was practiced lying down with mindful attention systematically scanning the body. Sitting meditation was practiced on a cushion or a chair, with either focused one-pointed attention (e.g. to the breathing) or with open monitoring of the constant changing flow of experience. Yoga consisted of dynamic movement in and out of certain poses, with continuous awareness of bodily sensations. Moreover, the weekly meetings consisted of group dialogues about both on-going practice and topics related to stress biology and stress reduction. The only minor deviation from the MBSR manual was a 20-min dialogue about CAD and stress in session 4. The MBSR teacher was at the time of the study enrolled in the second phase of MBSR teacher training, had 3 years of experience teaching MBSR, and led the CAD patient group under supervision from a certified MBSR supervisor.

### Data collection

Diary based methodologies can be particularly suitable when the research question is focused on exploring change over time [33]. Participants received a diary, developed by the research group, with extensive experience in the interdisciplinary research field of behavioural cardiology. The research group contained cardiologist, cardiac nurse, mindfulness instructor as well as experts in clinical psychology and qualitative methodology. The diary notebook contained written instructions about the narrating during the MBSR intervention, in which the participants were encouraged to write expressively and freely about their experiences for 5–15 min after each home practice session. If words did not seem to flow easily, they were encouraged to reflect over one or some of the following questions: *How did you feel during practice? Did any particular thoughts or stories appear? Did any particular emotions or moods occur? Was it pleasant or unpleasant to practice? How did you handle (the pleasant or unpleasant) experience? What are your feelings here and now after the practice session? Which thoughts appear now when you reflect over your practice session?* The development of these questions was inspired by the goals expressed in the MBSR manual, but since the analytical method was conventional and inductive we aimed at keeping the questions open and not linked to any theoretical framework. This non-directive focus on the immediate experiences of feelings, thoughts, moods and ways to handle the experiences, could reveal meaningful benefits from, and barriers to, the practice of meditation and yoga. Twelve participants, of the 16 who completed the course, filled out their diaries according to instructions, and all entries were included in the analysis. Among the four completers whose diaries were not included in the analysis, two was empty of written content, and two did not hand in their diaries at the end of the MBSR intervention.

### Ethical considerations

Systematic reviews of MBI:s have shown that these interventions have very few inherent dangers or potential side effect [18]. We were aware of the fact that participation without completion could be experienced as a failure, and perhaps worsen a sense of hopelessness. However, all patients had the opportunity to specifically address these issues with their assigned cardiac rehabilitation nurse. Participants were informed that the diaries would be collected at the end of the intervention and handled as a confidential document. We are not aware of any potential side effects of writing narrative entries in a diary, and since “journaling” are often encouraged as a complementary reflective contemplative practice during MBSR, the extra burden in time and energy were deemed reasonable. We anticipated that some participants might feel strong aversion against the writing assignment and we therefore added to the written instructions a statement that clarified that it was acceptable with very short reflections or sometimes nothing written at all to prevent a sense of pressure. Written informed consent forms were obtained from all participants prior to enrolment, and the local Ethical Review Board of Linköping gave its approval to the study (registration number: 2013/17/31).

### Data analysis

A qualitative method was applied to the analysis of the linguistic content in the diaries [34, 35]. The content analysis approach can be either conventional or directed, also described as inductive or deductive category development. In conventional content analysis, coding categories are derived directly from the text data. With a directed approach, according to Hsieh and Shannon, analysis starts with a theory or relevant research findings as guidance for initial codes [36]. As there was not enough previous research about the phenomenon, a qualitative, conventional approach was applied. The first author (OL), who at the time was both PhD-student in medicine, psychology student with a bachelor’s degree and intern physician, performed the first three steps in the analytic process independently. The first author (OL) had long personal, as well as teaching, experiences of mindfulness meditation. During the analysis, this pre-understanding was put aside to the largest extent as possible in order not to let it influence the interpretation of data. In the first step of analysis diary entries were transcribed into a word file with a total of 46 double-spaced pages of data and excerpts were tagged with a coded number as a way to prevent identification. The word file was then read and re-read multiple times to achieve immersion and obtain a sense of the whole. The focus was immediate experiences of mindfulness practice with the questions/prompts in the diaries guiding the analysis (see section data collection, above). Mostly, the diary entries were longer and more detailed in the first half of the course and shorter in the second half. In the second step quotations that appeared to capture key thoughts or concepts were highlighted in their exact words. A total number of 459 quotations were derived from the data. During this phase all relevant quotations were coded into more condensed sentences, and the *codes* were also tagged with a week-number (which one of the 8 weeks of MBSR) according to the date it was originally written. The resulting 122 codes could be read in the Additional file 1. The majority of the participants wrote free reflections while a few pondered the suggested questions. Then, in the third step, first impressions about the content in the codes were annotated as initial analysis, and codes were then grouped into *emergent subcategories* based on how the different codes were related and linked. These emergent subcategories were used to organize and group codes into *meaningful clusters*. In the fourth step, some overlapping was found and finally six *subcategories* were condensed into two *categories*. Both categories emerged concurrently over time and all participants’ experiences were represented in both categories. Lastly, in the fifth step, the categories were condensed into one more interpretative *main category,* to capture the time frame of the entries. Two examples of the analysis process are presented in Table 1. The analysis suggested that saturation of content variety was reached within our data after 10 diaries, since the last two diaries did not provide any new *codes.* The analysis was validated by checking for the representativeness of the data as a whole by thoroughly discussing the coding scheme, clusters and the preliminary categorisation with the co-authors who had extended experience in study design and clinical research (PG, MK, LJ) and qualitative content analysis (IT). Disagreements were discussed until consensus was reached. Finally, each category was strengthened by quotations. The quotations were translated from Swedish into English by the first author (OL), edited by a professional translator and then again read and compared with the original language by the co-authors.

**Table 1:** Examples of the analysis

Quotation →	Code →	Subcategory →	Category →	Main category
*It is hard, even impossible, to relax.* *At the same time, it fosters an understanding of how tense I am.*	Hard to relax and feeling tense	Struggling with bodily sensations	Facing the challenge of daily practice	*A journey through chaos and calmness*
*I am beginning to feel pretty good while practicing. And the best thing is that I feel energized afterwards – that is my reward*	Feeling pretty good and energized afterwards	Beginning to sense positive effects	Harvesting the fruits of daily practice	*A journey through chaos and calmness*

### Analytic rigour

Trustworthiness, defined as credibility, transferability, dependability and confirmability must be considered when evaluating qualitative data [37]. Credibility was established through ensuring the richness of the data by including participants, with rich experience of participating in an 8-week MBSR program, that were able and willing to share their immediate reflections in a diary. This method also allowed persistent observations over time. All participants that had filled out the diary were included in the analysis, which further increased credibility. To facilitate transferability, a clear description of the context, selection and characteristics of participants, data collection and process of analysis were presented. The procedure of data analysis was described in detail and a critical examination of the structure of the categories by all the authors were further steps to ensure dependability. Confirmability was achieved with the conventional (inductive) approach to content analysis, which grounds the analysis in the participant’s reflections. Confirmability was furthermore established with some of our findings converging with the existing literature.

## Results

Four women and eight men provided diary entries for the analysis. Background characteristics of the participants are shown in Table 2.

**Table 2:** Background characteristics of study participants, (*N* = 12)

	Median	IQR^b^
Age, years	62	56–63
Female/Male (n)	4/8	–
Index event^a^ (n)		
MI	10	
PCI	1	
CABG	1	
Working (y/no)	7/5	–
Retired (yes/no)	2/10	–
Smoking (yes/no)	1/11	–
Hypertension (yes/no)	11/1	–
Diabetes mellitus (yes/no)	2/10	–
Antidepressants (yes/no)	2/10	–
Depressive symptoms^c^		
Range 0–60	20	15–24
Anxiety^d^		
Range 0–21	8	5–11
Self-rated daily practice^e^		
Min per day	15	5–25

^a^*MI* myocardial infarction, *PCI* percutaneous coronary intervention, *CABG* coronary artery by-pass graft surgery

^b^*IQR* inter quartile range

^c^Centre for epidemiological studies depression scale (CES-D) prior to MBSR

^d^Generalized anxiety disorder 7 scale (GAD-7) prior to MBSR

^e^Assessed by self-report questionnaires after MBSR

The main category, categories and subcategories are described in Table 3. The proportions of diary entries written at the beginning (week 1–2), middle (week 3–6) and the end (week 7–8) of the course have been visualized in bars.

**Table 3:**
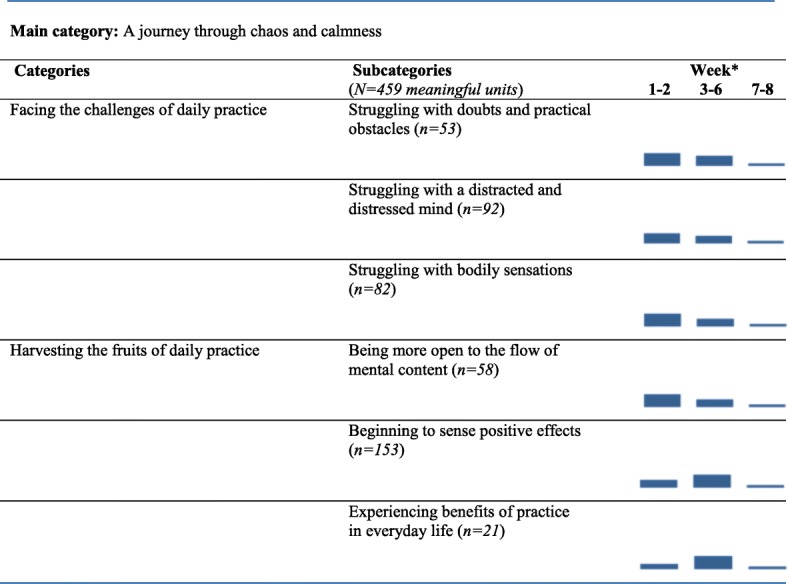
Findings

* Bars represents proportions of meaningful units written in the beginning (week 1-2), middle (week 3-6) and the end (week 7-8) of the MBSR course, in respective subcategory

### A journey through chaos and calmness

Taking on the challenge of daily mindfulness practice, the participants were describing a journey with obstacles and struggles, as well as rewarding experiences. This journey appears to reflect a progressive development culminating in the harvesting of the fruits of practice. The participants experienced both struggles and rewards continuously over time. Descriptions of various challenging facets of mindfulness practice, both physical and psychological, commonly occurred during the whole 8-week course, although distressing experiences were more predominant during the first half. The diary entries showed a wide variety of ways of dealing with these struggles, including both constructive and less constructive strategies of facing difficult experiences. As the weeks passed, the participants more frequently described an enhanced ability to concentrate, relax and deal with various distractions. They also put into words a heightened ability to observe the content of their mind and reported a number of ways the practice was starting to yield rewards in the form of positive feelings and a sense of mastery and well-being.

### Facing the challenges of daily practice

Facing the challenges of daily practice refers to how the participants struggled with obstacles to daily practice, with a distracted and distressed mind, as well as with bodily sensations.

### Struggling with doubts and practical obstacles

Especially during the first weeks of the course, the participants described various doubts and obstacles to daily practice. Two participants had difficulties understanding the meaning of the practice and two participants expressed doubt about their personal suitability for mindfulness. There were also notes from two participants about difficulties understanding the instructions and one patient expressed doubts about the right level of effort when practicing. A 63-year-old man reflected during the first week of practice:

*I wonder if I take this practice too lightly, but if this is the case, I guess I wouldn’t spend a whole hour trying.*

Many participants also felt stressed about finding the time to practice and two participants described the journaling as challenging. One participant also realized that it was hard to change ingrained behaviours and habits and three participants found it difficult to prioritize themselves.

### Struggling with a distracted and distressed mind

Eleven out of 12 participants described some kind of struggle with distractions and distressing feelings during practice session. They frequently reported becoming disturbed by sounds from the environment and also from uninvited mental content and impulses. A 62-year-old woman noticed during the second week:

*I was expected to be present here and now, but suddenly my thoughts were engaged in how to rearrange the curtains.*

Eight participants described feeling impatient, stressed, worried and unable to relax. Some noticed how they continuously judged their performance and subsequently felt a longing for signs of progress. A 63-year-old man wrote during the second week of the course:

*I would love to feel that I take the next step while doing this practice. But at the same time, I’m not sure what this step would mean.*

### Struggling with bodily sensations

All 12 participants described various physical symptoms and unpleasant sensations in the body during practice and two reported becoming aware of pain and tension that they had not noticed before. A 63-year-old woman wrote the following passage in her diary during the third week of training:

*When I think about it, I realize that I have aches in my body, all the time more or less. I haven’t thought about that before.*

Another related and frequently reported challenge was mental fatigue, drowsiness and a tendency to fall asleep, which were reported by seven participants. Two participants also described a sense of heaviness that emerged during practice. During the first couple of weeks three participants also noticed muscle soreness as a result of the yoga practice.

### Harvesting the fruits of daily practice

Harvesting the fruits of daily practice refers to how the participants became more open to the flow of mental content and begun to sense positive effects as well as benefits of practice in everyday life.

### Being more open to the flow of mental content

Five participants described an increased ability to observe the flow of thoughts and sensations during practice. These patients became more aware of the continuously changing stream of experiences and five participants noticed an altered sense of time. During the end of the second week, a 76-year-old woman wrote in her diary:

*I am doing the sitting meditation, focusing on my breathing, my nose, my chest, my belly. I listen, really listen, and now I am there, almost all the time. I’m starting to get what this is all about.*

Two participants also described a positive sense of emptiness. A 63-year-old man commented, at the end of the third week, on his just finished body scan practice:

*At the beginning, the thoughts set off in different directions, but along the way it got better and at times I got this feeling of “emptiness”; like I was entering another world.*

### Beginning to sense positive effects

Eleven out of 12 participants found it increasingly easier to deal with distractions and two of them clearly expressed a positive feeling when they, as part of the mindfulness technique, managed to return their awareness to their chosen object of meditation. A 63-year-old man described this experience, occurring during the fourth week, with a fragrance of accomplishment:

*My thoughts set off sometimes but I am trying not to get irritated and instead just trying to come back to the right feeling. Instead I try to think that it is a good thing that I managed to come back to the right feeling and praise myself. I tried to think that when I become distracted it is ok. Instead I do well when I bring back the right kind of focus. It seemed like this was helpful.*

Parallel to the continuous struggles, participants more frequently began to describe positive effects, both during and after the practice sessions. Six participants expressed feeling calm and relaxed while seven participants reported feeling energized after a meditation session. A 66-year-old man commented on a yoga session during week five:

*These practices, when I get to stretch my body, feel good and I think that I am smoother in my joints afterwards, but also, I sense a calmness in my soul.*

Six participants also described unpleasant sensations in a positive framework that might be related to the purpose of the mindfulness practice. A 57-year old woman wrote immediately after a yoga session the third week:

*You feel stiff, and it aches and crackles in the joints, but somehow it feels good anyway to stretch out on the mat. Forgot time. A bit of headache afterwards.*

### Experiencing benefits of practice in everyday life

At some time point during the course, eight out of 12 participants expressed a realization that the mindfulness practices, although sometimes hard to do, did produce tangible pay offs in daily life. A 63-year-old woman described a new insight with the following words:

*I begin to wonder if I have begun to think a little bit differently? It seems like I don’t ruminate as much – we’ll see.*

Several participants wrote in their diaries that they found themselves having more patience with life and that they could deal more effectively with stress. Three participants described how the mindfulness practice had made them more sensitive to the aliveness of their natural surroundings, and two participants seemed to feel empowered by the discovery that presence could have a calming effect on turbulent emotions.

The experiences described were both universal and highly individual processes and this was most apparent in the various diary entries written after the silent day at week 6. A 57-year-old woman wrote in her journal:

*The silent day was a different experience. Restful, inspiring, relaxing and it softened the body and the soul in a calm way.*

A 47-year-old man described the experience of the whole day in silence in very different words:

*The time flew away and as usual I did not feel much at all during the practices. At the end of the day, though, I experienced a kind of depressive feeling.*

## Discussion

We set out to explore the potential benefits from, and barriers to, the practice of mindfulness meditation through content analysis of diary entries. Our aim was to describe the immediate experiences of practice among CAD patients with depressive symptoms after a recent coronary event. The journey of MBSR was characterized by the simultaneous and continuous occurrence of struggles and rewarding experiences, although we also noticed that the struggles were predominantly occurring during the early phase of the course. Our findings suggest that this dynamic interplay between struggles and rewards, and the attempts to deal constructively with it all, may underlie the strengthened skills of focused attention, openhearted embrace of experience and increased psychological flexibility that characterize the phenomenon *mindfulness*. This interpretation is supported by theoretical frameworks of the wholesome potential in facing difficulties and distractions with a curious, open and non-judgmental mind [38, 39].

Facing challenges was a prominent feature of participants’ diaries, but this aspect of mindfulness practice has not gained the same attention in earlier studies of participants’ experiences as more positive aspects. In a summary of 14 qualitative studies of MBI:s, Malpass et al. [40] made a synthesis of the therapeutic process in mindfulness. The only description of struggles is the facet “facing the difficult” in their final model. Morone et al. [41] used content analysis of diary entries in their study of older adults with chronic pain, participating in MBSR. They report themes associated with pain reduction as well as experienced improvements in attention skills, sleep, well-being, but also difficulties in finding the time to practice and becoming sleepy*.*

Likewise, Griffith et al. [26] who studied CAD patients after MBCT, reported almost exclusively positive experiences, with the minor exception of the findings that some patients were struggling with the body scan practice. In line with this, Mason et al. [42] reported mostly positive experiences in their study of depressed patients in MBCT, even if their results also included the subcategory initial negative experience. On the other hand, an earlier study of Swedish cancer patients, using semi-structured interviews and thematic analysis, reported that participants also had negative experiences associated with the meditation- and yoga practices [43].

Mindfulness teachers often inform their students that to just sit and pay attention to the breath can be surprisingly challenging [17]. Our findings further elucidate this by describing in depth the experience, and the continuous nature of this struggle, what the participants struggle with, and also what it feels like. This knowledge could be of importance for how future participants are prepared for mindfulness training. Realistic expectations could boost motivation and perseverance in ways that are helpful during the challenging early phases of mindfulness training.

It is important to bear in mind that the participants in our study, with a history of a recent CAD event, were selected on the basis of having subclinical or mild clinical depression. These two characteristics might have caused a rougher journey with higher loads of both psychological distress and physical symptoms to deal with. However, during the analysis and categorization of data, references to depressive symptoms as well as CAD events were surprisingly few. One way to interpret this finding is that depressive symptoms may contain a diverse ensemble of facets [30] and thus hide behind the surface of the more universal struggles. Indeed, part of the content in our analysis could be viewed as facets of depressive symptomatology, but it is also apparent that many of these experiences represent common facets of the human predicament with its universal hardships [44]. Perhaps, seeing this universality of distress can help the patient to avoid unnecessary and self-centred rumination [45]. Regarding the few narratives to the CAD diagnosis, it is one possibility among many that the mindfulness practices – with its focus on non-conceptual awareness of the immediate experience of being human – could have given the participants a wholesome pause from the habitual identification with their role as CAD patients [39]. This is in line with the proposed mechanisms of the salutary effects of mindfulness training in which non-identification with views of self and others is proposed as a kind of final step in the complex process of psychological change initiated by mindfulness practice [39]. van der Velden et al. [46] conducted a systematic review of mechanisms involved in the effects of mindfulness training. They showed that changes in worry and rumination, as well as mindfulness skills, and possibly also factors of attention and emotional reactivity, mediated the positive effects [46]. There is apparently a large convergence between these proposed mechanisms and the written content in the diaries of our participants. This convergence confirms that the combination of a history of previous CAD event and persistent depressive symptom does not provide barriers to participation in and gains from the MBSR intervention. This conclusion may be of interest for healthcare providers who consider mindfulness-based stress reduction as an alternative to other psychosocial interventions in the context of cardiac rehabilitation.

Hölzel et al. [39] proposed that emotional regulation skills improve through continuous *exposure* to challenging sensations, and when faced with openness and curiosity, this may lead to the *extinction* of conditioned habitual emotional reactions. In one of the first diary-based studies of participants’ experiences during mindfulness practice, Kerr et al. [47] showed that participants developed an *observing attitude* towards their own distress. Our findings that participants are becoming increasingly more open to the flow of sensations and thoughts, and that this progress might be related to the improvement of functioning in daily life, are thus in line with these earlier findings.

### Methodological considerations

The use of diary entries written in immediate proximity to the practice sessions has inherent strengths and limitations. The closeness in time between lived experience and written reflection and the continuous collection of entries during the whole 8-week course are two key strengths of this method. This has enabled us to get a more nuanced picture of participants’ experiences as well as information of how the process of participation unfolds over time. Furthermore, there might be less risk of bias from participants’ desire to please and accommodate to the researcher compared to an interview. Our data captured the continuous struggles, which might have been partly forgotten (or repressed) months after completion of the course. Based on this, we argue that this particular kind of qualitative methodology may facilitate a critical examination of the role for mindfulness-based interventions in healthcare practice. Our selected method does, however, constrain the depths of participants’ accounts of their experience. It prevents researchers from asking clarifying follow-up questions to particularly interesting answers. Furthermore, our participants were more eager to write in their diaries during the first half of the course, thus conclusions drawn from the descriptive analysis of the time points for the diary entries should be made with caution. Another important limitation is the selection of study population since all of our participants were completers of the entire MBSR-course. It is possible that dropouts had similar experiences of struggles and distress, and hence it would have been interesting to also examine whether dropouts reacted differently. This question should be addressed in future studies since adherence to practice and completion of mindfulness interventions are well-known challenges in the work of implementing this method in clinical practice. The moderately high dropout rate from the intervention, and the failure of 4 completers to adhere to the writing instructions, provided limitation on the amount of data available for analysis. However, the data from 10 out of our 12 participants with full participation and available diaries did reach saturation in content.

## Conclusions

In conclusion, we have found that mindfulness training among patients with depressive symptoms after a recent CAD event is a tough and challenging, but also manageable and potentially fruitful, endeavour. Furthermore, we suggest that the dynamic co-occurrence of struggles and rewards can promote mindfulness skills and new ways to relate to distressful experiences. The findings highlight and describe various challenges inherent in mindfulness practices. They also suggest that MBSR-participants need motivational support and skilful guidance throughout the whole course. Moreover, our findings indicate that teachers and participants need to entertain realistic expectations if the journey through chaos and calmness is to bear fruit among those who accept the challenge.

## Additional file

Additional file 1: Codes derived from meaning units in raw data. Contains codes (short sentences) derived and condensed from the original raw data of participant’s diary entries. (PDF 44 kb)

## Abbreviations

CABG: Coronary artery by-pass graft surgery
CAD: Coronary artery disease
CES-D: Centre for epidemiological studies depression scale
IQR: Inter quartile range
MBI:s: Mindfulness based interventions
MBSR: Mindfulness based stress reduction MBCT Mindfulness based cognitive therapy
MI: Myocardial infarction
PCI: Percutaneous coronary intervention
