# Supplementary material for: A journey through chaos and calmness: experiences of mindfulness training in patients with depressive symptoms after a recent coronary event - a qualitative diary content analysis
Source: BMC Psychol. 2018 Sep 13;6:46. doi: 10.1186/s40359-018-0252-1 (PMC6136221; doi:10.1186/s40359-018-0252-1)
Supplement: Supplementary file 1 — Codes derived from meaning units in raw data. Contains codes (short sentences) derived and condensed from the original raw data of participant’s diary entries. (PDF 44 kb) [file 40359_2018_252_MOESM1_ESM.pdf]

Supplemental Table: Codes derived from meaning units in raw data

Difficulties getting the point of practice
Realizing it is hard change behaviours
Reflecting over expectations
Reflections about how hard it is to prioritize oneself
Having doubts about suitability for mindfulness
Reflecting thoughts about the instructions
Difficulties understanding the practices
Having doubts about the right level of effort
Reflections over how to get the technique right
Reflecting over practicing in different places and times
Feeling stressed about finding the time to practice
Finding it hard to write in the journal
A sense of starting from the beginning all over again
Doubts about capacity to deal with Silent day
Realizing unwholesome ways to cope with negative experiences
Struggling with disturbances from the surrounding environment
Struggling with unpleasant and distracting thoughts during practice
Difficulties concentrating while practicing
Difficulties relaxing during practice
Feeling stressed and worried during practice
Feeling impatient during the practice
Feeling a longing for signs of progress
Experiencing a lack of healing closeness to nature
Judging the practice performance
Comparing different practices with each other
Comparing experiences with earlier sessions
Observing judging attitude and correcting ones attitude
Feeling depressed by slow walking
Feeling depressed after the Silent day
Feeling sadness during Silent day, and realizing a need for closure
Experiencing a sense of sensory overload during walking
Feeling disappointment over the practice experience
Realizing existing pain and aches has been there before
Experiencing unpleasant sensations and physical symptoms
Feeling tired during practice
Falling asleep – or almost – during practice
Reflecting over strategies for dealing with drowsiness
Experiencing unpleasant sensations after the practice
Feeling irritation after the practice
Experience of muscle soreness afterwards
Observing a stream of different sensations during practice
Observing the breathing during practice
Observing neutral physical sensations during practice
Noticing bodily tensions from everyday experiences
Observing neutral experiences of sounds in the environment
Observing the flow of thoughts during practice
Reflections over quietness
Experiencing not much at all during practice
Experiencing no special results from practicing
Experiencing surprising and unknown mental content
Experiencing an altered sense of time passing
Experiencing a sense of heaviness during practice
Experiencing surprising physical sensations during practice
Experiencing various senses of emptiness during practice
Hard to focus but hopeful anyway
Difficulties at start but an experience o flow after a while
Noticing simple yoga poses can be challenging
Starting to realize what's the point of practicing
Finding one dealing better with distractions during practice
Finding it easier to concentrate and feeling a sense of progress
Experiencing a sense of flow while practicing without guidance
Reframing unpleasant sensations and difficulties as a positive experience
Experiencing the lying down as pleasant
Feeling calmness, lightness and relaxation during practice
Experiencing less unpleasant sensations during practice
Feeling calmer after practice
Feeling more relaxed after practice
Feeling energized after practice
Experiencing longing for the next practice session
Feeling relaxed after the Silent day
Experiencing elation and energy and an subsequent emotional dip
Experiencing the Silent day as pleasant and rewarding
Finding it easier to be present and find a calmness during the Silent day
Having patience with the whole process
Observing a stream of different sensations during practice
Observing the breathing during practice
Observing neutral physical sensations during practice
Noticing bodily tensions from everyday experiences
Observing neutral experiences of sounds in the environment
Observing the flow of thoughts during practice
Reflections over quietness
Experiencing not much at all during practice
Experiencing no special results from practicing
Experiencing surprising and unknown mental content
Experiencing an altered sense of time passing
Experiencing a sense of heaviness during practice
Experiencing surprising physical sensations during practice
Experiencing various senses of emptiness during practice
Hard to focus but hopeful anyway
Difficulties at start but an experience o flow after a while
Noticing simple yoga poses can be challenging
Starting to realize what's the point of practicing
Finding one dealing better with distractions during practice
Finding it easier to concentrate and experiencing a sense of progress
Experiencing a sense of flow while practicing without guidance
Reframing unpleasant sensations and difficulties as a positive experience
Finding rewarding feelings when awareness returns to object of meditation
Experiencing mindful movement as rewarding
Experiencing the lying down as pleasant
Feeling calmness, lightness and relaxation during practice
Experiencing less unpleasant sensations during practice
Feeling calmer after practice
Feeling more relaxed after practice
Feeling energized after practice
Experiencing longing for the next practice session
Feeling relaxed after the Silent day
Experiencing elation and energy and an subsequent emotional dip
Experiencing the Silent day as pleasant and rewarding
Finding it easier to be present and find a calmness during the Silent day
Having patience with the whole process
Realizing that presence has a calming effect
Experiencing more patience with life
Experiencing less resistance in everyday life
Experiencing less rumination in everyday life
Experiencing a heightened awareness of nature
Experiencing a heightened awareness of thoughts and reactions during stress
Experiencing a sense of flexibility and control in life
Finding it easier to deal with stress at work
Finding the meditation useful in dealing with anxiety and tension
Finding it easier and rewarding to reach out to others
Reflecting over mindfulness as an integrated habit in life
Embracing difficult experiences as a kind of therapeutic process
Mindfulness as part of a larger process of change
